# Rapid transition from primary to secondary crust building on the Moon explained by mantle overturn

**DOI:** 10.1038/s41467-023-40751-7

**Published:** 2023-08-17

**Authors:** Tabb C. Prissel, Nan Zhang, Colin R. M. Jackson, Haoyuan Li

**Affiliations:** 1 NASA Johnson Space Center, Astromaterials Research and Exploration Science Division, 2101 NASA Parkway, MailCode XI3, Houston, TX 77058 USA; 2 Key Laboratory of Orogenic Belts and Crustal Evolution, School of Earth and Space Sciences, Peking University, Beijing, 100871 China; 3 School of Earth and Planetary Sciences, Curtin University, GPO Box U1987, Bentley, WA 6845 Australia; 4 Department of Earth and Environmental Sciences, Tulane University, 6823 St. Charles Avenue, New Orleans, LA 70118-5698 USA; 5 Department of Earth and Planetary Sciences, University of California, Davis, One Shields Avenue, Davis, CA 95616 USA

**Keywords:** Geodynamics, Petrology, Inner planets, Geochemistry, Meteoritics

## Abstract

Geochronology indicates a rapid transition (tens of Myrs) from primary to secondary crust building on the Moon. The processes responsible for initiating secondary magmatism, however, remain in debate. Here we test the hypothesis that the earliest secondary crust (Mg-suite) formed as a direct consequence of density-driven mantle overturn, and advance 3D mantle convection models to quantify the resulting extent of lower mantle melting. Our modeling demonstrates that overturn of thin ilmenite-bearing cumulates ≤ 100 km triggers a rapid and short-lived episode of lower mantle melting which explains the key volume, geochronological, and spatial characteristics of early secondary crust building without contributions from other energy sources, namely KREEP (potassium, rare earth elements, phosphorus, radiogenic U, Th). Observations of globally distributed Mg-suite eliminate degree-1 overturn scenarios. We propose that gravitational instabilities in magma ocean cumulate piles are major driving forces for the onset of mantle convection and secondary crust building on differentiated bodies.

## Abstract (web summary)

Overturn of late stage lunar magma ocean cumulates triggers a rapid & short-lived episode of lower mantle melting that explains the key volume, geochronological, & spatial characteristics of the earliest secondary crust on the Moon (Mg-suite).

## Introduction

Akin to the theory of plate tectonics on Earth, the magma ocean and cumulate mantle overturn (CMO) hypotheses work in concert as the guiding paradigms for the formation and redistribution of mantle and crustal material on terrestrial bodies^1–3^. These concepts were largely developed through exploration of the Moon, and its rock record still provides the most direct evidence for magma ocean and CMO epochs. Here the lunar magnesian-suite of samples stand out (Mg-suite: dunite, pink spinel troctolite, troctolite, norite, gabbronorite). Their forsteritic olivine composition anchors the Mg-suite mantle source to initially deep-seated lunar magma ocean (LMO) cumulates, and their presence within the primary lunar crust demands mobilization of said deep-seated cumulates toward the surface via CMO^3–7^. Geochronology further indicates that Mg-suite petrogenesis, and by extension possibly CMO, occurred near-contemporaneously with primary lunar crust solidification^8,9^. Thus, the Mg-suite plays a pivotal role in unraveling the magmatic transition from primary to secondary crust building on the Moon. Despite these critical links to early lunar evolution, a lack of consensus remains regarding the operative mechanisms responsible for generation of early secondary magmas and their global extent^10–13^.

The Mg-suite samples returned by the Apollo missions are confounding because they contain elevated concentrations of incompatible elements thought to be associated with a KREEP component (potassium, rare earth elements, phosphorus)^5–10^. The KREEP signature observed in Mg-suite samples is surprising because the formation of KREEP is tied to the final stages of LMO crystallization, contrasting with the primitive origins demanded by their major element chemistry. Determining the role of KREEP during Mg-suite petrogenesis is important because its high concentrations of U, Th, and K make KREEP a major source for radiogenic heat in the magmatic evolution of the Moon^14,15^. KREEP-induced melting was recently proposed to be the primary mechanism for explaining the observed lunar crustal dichotomy^12^, potentially determining the production and distribution of Mg-suite magmatism^5,6^.

Mounting lines of evidence now call into question the importance of KREEP during Mg-suite petrogenesis. KREEP-poor lunar meteorites with a chemical affinity to Mg-suite are documented^10,16–20^, geochemical models demonstrate no need for KREEP to produce Mg-suite parental melts derived from primary LMO cumulates^11^, and remote sensing observations identify candidate Mg-suite locations across the lunar surface^21–23^, far beyond the Procellarum KREEP Terrane (PKT) where KREEP appears most concentrated. If KREEP is not a primary driver of Mg-suite petrogenesis, CMO would rise as a central geologic process for initiating secondary crust building on the Moon.

KREEP-free geochemical links between Mg-suite and CMO have been recently forwarded^8,11,16^, and modern dynamical models of early mantle convection^24^ identify overturn timing as a critical component to cementing a CMO origin for Mg-suite. However, the abundance, timing, and spatial extent of lower mantle melting during CMO has yet to be fully quantified. Moreover, the last decade has delivered advances in both geochronology and global mineralogical analysis of the lunar crust that present new challenges to the CMO hypothesis and place new constraints on Mg-suite petrogenesis.

First, geochronological work identifies concordant dates for putative primary lunar crust and secondary Mg-suite samples^8,9,25–30^. Primary crust samples 60025, 62237, and lunar anorthositic meteorite Yamato 86032 provide a weighted average age of 4361 ± 21 Ma from the Sm-Nd isotopic system. Concordance with multiple chronometers (including ^147^Sm/^143^Nd, ^146^Sm/^142^Nd, and Pb-Pb) has been established for 60025, which yields a tightly constrained age of 4360 ± 3 Ma. The most reliable ages determined for secondary Mg-suite samples 15445 (4332 ± 79 Ma), 67667 (4349 ± 31 Ma), and 78238 (4334 ± 34 Ma) obtained via the Sm-Nd isotopic system yield a median of 4340 ± 9 Ma^8,25,30^. Samples 67667 and 78238 also yield concordant Rb-Sr ages (4368 ± 67 Ma and 4359 ± 24 Ma, respectively)^8,25^ and 78238 further yields a concordant ^207^Pb/^206^Pb age (4332 ± 18 Ma)^9^, indicating a record of magmatic emplacement and crystallization. These ages are concordant with the whole rock isochron age for the Mg-suite of 4348 ± 25 Ma, which includes samples from Apollo 14-17^8,30^. The dataset for both ferroan anorthosites (FAN) and Mg-suite samples is small and emphasizes the need for future geochronological investigations and additional sample return missions. Nevertheless, petrologic context requires that the primary lunar crust formed prior to secondary Mg-suite intrusions, and the most robust data above imply these two events were separated by only tens of millions of years or less. Using the weighted average age of FAN and the whole rock Mg-suite isochron above, the maximum disparity between FAN (4361 + 21 = 4382 Ma) and Mg-suite (4348 − 25 = 4323 Ma) dictates that CMO-driven origin models must produce secondary crust building within ~59 Myrs after primary crust formation. Further, the small variance associated with the whole rock isochron Mg-suite age itself (± 25 Myrs) requires that initial secondary crust building was short-lived, or ≤ 50 Myrs in duration.

**Fig. 1:**
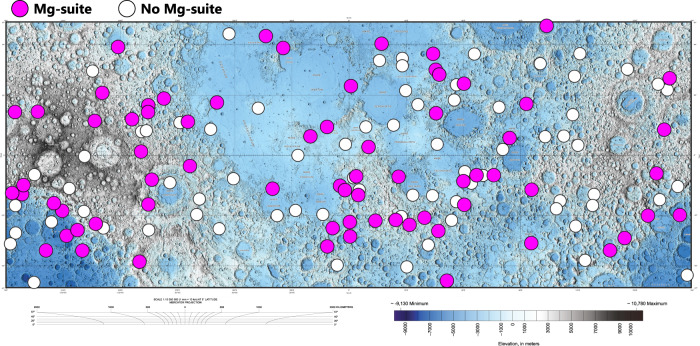
Global extent of candidate Mg-suite exposures. Edited topographic base map of the Moon published by the U.S. Geological Survey^91^. Mercator projection centered at 0^o^ longitude and between latitudes ± 57°. Color elevation scale provided. Pink-filled circles represent candidate Mg-suite detections (pink spinel, olivine, orthopyroxene) and white-filled circles are craters examined with no discernable detection of Mg-suite, all from the orbital remote sensing of 164 fresh and undisturbed crater central peaks across the surface of the Moon^21^.

Second, combined petrological and reflectance spectroscopy studies have linked orbital detections of pink spinel anorthosites from M^3^ data (Moon Mineralogy Mapper) to Mg-suite samples^21–23,31–35^. Outcrops of pink spinel anorthosites along with olivine- and orthopyroxene-rich exposures (major mafic constituents of Mg-suite rocks) are observed in fresh and undisturbed crater central peaks across the lunar surface^21–23^, indicating excavation of pre-existing crustal material. From these studies, the Mg-suite appears to be broadly distributed across the Moon and not isolated within a single regional terrane (Fig. 1). The presence of KREEP-poor meteorites with a chemical affinity to Mg-suite^10,16–20^, likely sourced from localities outside of the PKT, provide ground-truth to the global extent observed remotely. Although Mg-suite rocks appear widespread, they are estimated to comprise ~6–30 vol.% of the total lunar crust^21,36^ based on Clementine data and global investigations of crater central peaks containing troctolite, norite, and gabbronorite lithologies (predominant subgroups of the Mg-suite). The limited abundance of Mg-suite lithologies in the lunar crust therefore constrains the extent of melting in associated petrogenetic models.

Taken together, the emerging picture is that the Mg-suite formed near contemporaneously with primary FAN production during a short magmatic interval, is broadly distributed across the Moon, and constitutes a modest fraction of the lunar crust. Here we employ a modern three-dimensional mantle convection model^37^ to examine the spatial and temporal aspects of mantle melting produced by the upwelling return flow of primary magma ocean cumulates in response to CMO. We advance the existing geodynamic model by quantifying the timing and extent of lower mantle melting and integrating available data from geochronology, petrologic studies, and orbital spacecraft, to determine if (i) CMO-induced decompression melting of the lower mantle is capable of producing sufficient volumes of Mg-suite material, (ii) the magmatic duration of CMO-induced melting is consistent with the small variance observed in the most reliable Mg-suite crystallization ages, and (iii) the onset of CMO-induced melting can reconcile the apparent rapid transition from primary to secondary crust formation on the Moon. The spatial distribution of melting is then evaluated to test whether (iv) a CMO origin can simultaneously satisfy the observed extent of global Mg-suite exposures. In so doing, we identify physical properties of lunar CMO that ultimately satisfy modern observations of early secondary crust building.

## Results and discussion

We investigate the thermochemical evolution of density-driven cumulate mantle overturn and convective return flow of the lower mantle using a numerical three-dimensional model of spherical geometry^37^ and test the effects of ilmenite-bearing cumulate (IBC) thickness and viscosity contrast between the IBC layer and underlying mantle. Each simulation begins with a model Moon consisting of five layers from bottom to top: core, lower mantle (Mg-suite source), upper mantle, IBC layer, and crust. Our lower mantle is ~3% denser than the upper mantle (Supplementary Table S1) considering the relative mean densities between dunitic (lower mantle) and harzburgitic (upper mantle) phase proportions and their decreasing pressure of formation during magma ocean crystallization^3,38–40^. The IBC layer has density = 3460–3700 kg m^-3^ with viscosity up to 4 orders of magnitude lower than the underlying mantle^37,39,41–43^ and is overlain by a less dense crust. Overturn of our initial stratigraphy is induced via random distribution of chemical tracers^44^, meaning we assign no initial perturbation to the IBC-mantle interface. Following precedent^37^, our primary dataset (Runs 1–11) assumes an initial temperature profile equivalent to the peridotite solidus. The peridotite solidus also approximates the calculated effective solidus (~1647 °C at 4 GPa)^11^ of the experimentally determined^6^ bulk lunar lower mantle (Mg-suite source), which is further consistent with calculated mantle potential temperatures (>1600 °C) at the time of Mg-suite formation^45^. For these reasons the peridotite solidus serves as both our initial temperature profile of the mantle and its effective solidus in Runs 1–11. The local production of lower mantle melting in response to IBC-driven cumulate overturn is then solved using parameterized equations benchmarked by previous work^46^. Additional runs were performed testing the effects of both cooler and hotter initial temperature profiles on magmatic timing and melt volume, and these are also summarized below. Further details of our model inputs and justifications for explored parameter space are included in our supplementary information.

### Natural observations and constraints

Results are assessed using the following constraints to determine which models are most consistent with the natural observations.

**Fig. 2:**
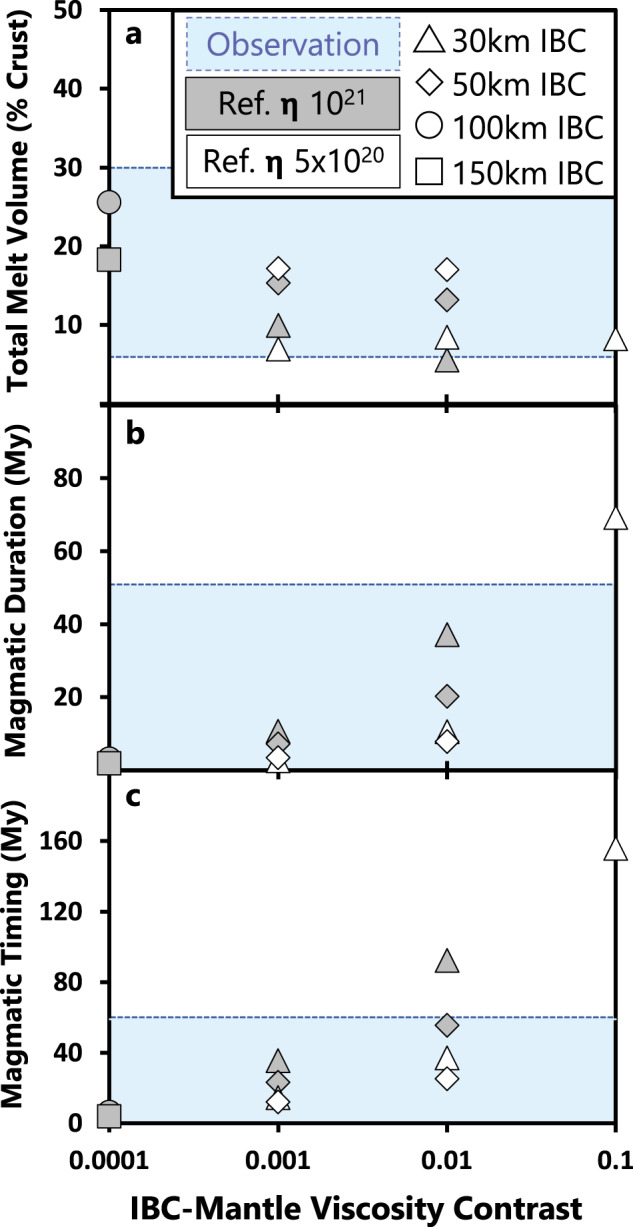
Melt volume and temporal systematics of lower mantle melting in response to cumulate mantle overturn. **a** Total melt volume derived from decompression melting of the lower mantle during mantle overturn, **b** the full width at half maximum of peak melt production, and **c** time to 50% cumulative melt volume, all plotted as a function of IBC viscosity contrast. Natural constraints (defined in the main text) are represented by blue-shaded regions and a legend is provided with reference viscosity given in Pa s. In general, the natural observations are well-explained by decompression melting of the lower mantle (Mg-suite source) in response to cumulate overturn, particularly when considering a low mantle reference viscosity of 5 ×10^20^ Pa s and a viscosity contrast of 10^-2^ or greater.
- Constraint 2 (magmatic duration) is defined by the estimated duration of Mg-suite magmatism based on concordant dating of Mg-suite samples. Here we define the magmatic duration of Mg-suite using the variance of the whole rock isochron (±25 Myrs^8,30^), or ≤50 Myrs duration (Fig. 2b). We approximate the duration of mantle melting in our dynamical models by measuring the full width at half maximum (FWHM) of peak melt production rates for each run (supplementary Fig. S1).
- Constraint 3 (magmatic timing) considers the interval of time between primary and secondary crust building. This constraint is defined by the maximum disparity between primary FAN and secondary Mg-suite ages (including their variance), or ~59 Myrs^8,30^ (Fig. 2c). In this study, we define the magmatic timing for each dynamical scenario as the interval between time zero of the model and the time step most closely associated with 50% cumulative melt volume derived from the lower mantle (Supplementary Fig. S2). The time to 50% cumulative melt volume therefore provides a relatively conservative estimate for the magmatic timing compared to the onset of melting for each run.
- Constraint 4 (exposure proportion) accounts for the detectability of Mg-suite rocks in craters across the lunar surface. Of 164 fresh and undisturbed crater central peaks examined with M^3^ data^23^, 85 contained evidence for Mg-suite material. Criteria for Mg-suite material was defined as multiple observations of pink spinel or olivine (or both) using multi-temporal images, and/or the observation of orthopyroxene in the absence of clinopyroxene (a potential marker for mare basalts). Given that 85 out of 164 total central peaks contained spectral signatures consistent with Mg-suite material, we determined an exposure proportion of 0.52 for the lunar surface. Our selection of this dataset^23^ is based on their extensive search of craters across the lunar surface that have excavated pre-existing crustal material (i.e., not impact melts) ranging from near-surface depths to the crust-mantle boundary, and their use of a common approach for mineral identification. Our model does not capture magmatic emplacement depths, but previous work^34^ demonstrated that Mg-suite primary melts can reach levels of neutral buoyancy throughout the crust, consistent with the remote identifications used here. To make the comparison between natural observation and model, we randomly sample the surface of our models 164 times to replicate the number of craters investigated. Each sampling location is a synthetic crater, and the area sampled by the synthetic crater is determined by the scaling relationship between central peak and crater diameter^48^ using the diameters of the craters reported^23^. If melt from the lower mantle is present in a sampled area, we tally an identification of Mg-suite (Fig. 3). A thousand iterations are performed with 164 randomized cratering locations that define an average synthetic exposure proportion. Our model includes a 2% melt detection threshold, which means that melting ≥ 2% is sufficient to be extracted from the source, mobilized toward the surface, and remotely detected. This is supported by constraints for the melt fraction retained in the source matrix during melting, which is unlikely to exceed 3% at any given time^49^. We note, however, that small increases in degree of melting will cause large increases in rock permeability^50^, which can result in the channelized flow of partial melts^51,52^. To account for this phenomenon in our spatial analysis, we increase the melt detection threshold (MDT) in 1% increments (up to 7% to remain within the partial melting constraints defined by geochemical modeling^11,12^) and run the same 1000 random cratering iterations for each percentage step. The resulting data can be taken to evaluate the spatial effects on global melt distribution within a system of low degree (MDT = 2–3%), moderate (MDT = 4-5%), and higher degrees of partial melting (MDT = 6–7%). Since the total melt fraction retained in the matrix during partial melting is not expected to be > 3% at any given time, our model assumes that the total melt volumes are not significantly changed with increasing MDT (i.e., the total amount of escaped melt is merely channelized into areas of increased permeability).

- Constraint 1 (Mg-suite volume) is defined by the estimated amount of Mg-suite material within the lunar crust, or ~6–30 vol.% of the total lunar crust^21,36^. The total volume of decompression melt derived from the lower mantle during IBC-driven cumulate overturn is then converted to volume percent of the lunar crust to compare with the natural observations (Fig. 2a). The reference volume of the lunar crust is estimated by assuming a spherical shell and using a crustal thickness of 40 km^47^.

**Fig. 3:**
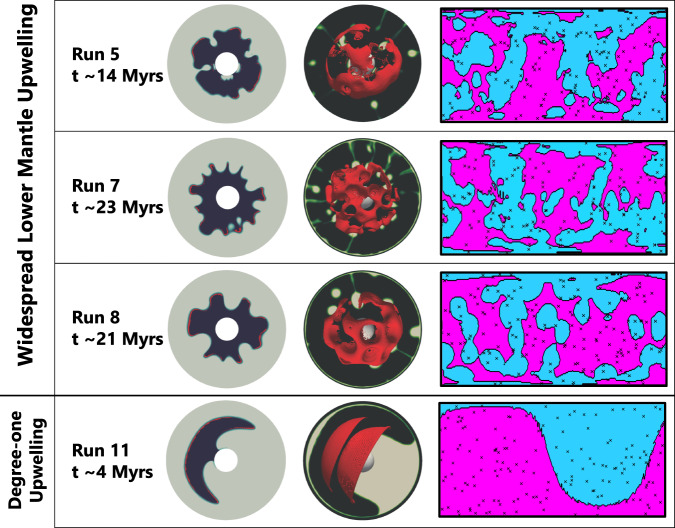
Morphology and melting of upwelling lower mantle in response to cumulate mantle overturn. Runs 5 (IBC = 30 km), 7 and 8 (IBC = 50 km), and 11 (IBC = 150 km) are showcased. Presented in each row are snap shots of model runs near peak melt production. Left: isolating the 2D cross-section morphology of upwelling lower mantle (Mg-suite source) in navy blue relative to all other interior components (light gray) and associated regions of decompression partial melting are highlighted in red. Middle: visualization of the 3D melt surface from upwelling lower mantle (red) overlaying an isolated 2D slice of the downwelling IBC (yellow-green to gray) relative to all other interior components (black). Right**:** the surface expression of the 3D melt surface considering a melt detection threshold of 4% with regions of partial melting (pink), no melting (blue), and synthetic crater locations (x) used to determine exposure proportions and farthest neighboring distances (see also Fig. 4). Runs 5, 7, and 8 highlight that widespread lower mantle upwelling patterns are common during cumulate overturn (additional cases are shown in Supplementary Fig. S3). Run 11 is the only model that was dominated by a spherical harmonic degree of 1 for lower mantle upwelling.
- Constraint 5 (farthest neighboring detection) considers the total spatial distribution of Mg-suite rocks across the lunar surface. Although the complete distribution of subsurface Mg-suite is unknown, the observed spatial distribution of Mg-suite exposures can be quantified by measuring the current distance between each detection and its farthest neighboring detection. If Mg-suite detections are confined to a small region of the Moon for example, the farthest neighbor distance for each detection will be relatively short compared to the farthest neighbor distance of globally distributed Mg-suite locations. We calculate the average farthest neighbor of observed Mg-suite exposures^23^ to be 5103 ± 243 km, which is nearly half the circumference of the Moon (~5460 km), or the maximum farthest neighbor distance achievable. Further, we report the average nearest neighbor distance to be 266 ± 246 km. The high variance associated with the average nearest neighbor distance indicates a widespread distribution (as is visually observed) and is inconsistent with a regional cluster of exposures (Fig. 1). The average farthest neighboring distance in our synthetic crater model is measured the same way as the natural observations for comparison.

### Total melt volume derived from the upwelling lower mantle

All Runs 1–11 successfully meet Constraint 1. Downwelling of thicker IBC layers generally leads to greater total melt volume derived from the responsive upwelling of the lower mantle (Fig. 2a). The IBC-mantle viscosity contrast (hereafter, viscosity contrast) does not systematically correlate with total melt volume (Fig. 2a). Model runs with IBC thicknesses of 30 km (Runs 1–5) yield total melt volumes ranging from 6–10 vol.% of the lunar crust, whereas runs with IBC thicknesses of 50 km (Runs 6–9) yield 13–17 vol.% (Table 1). Runs 10 (IBC = 100 km) and 11 (IBC = 150 km) resulted in melt volumes proportional to 26 and 18 vol.% of the lunar crust, respectively. We note that the total melt volumes reported here are a conservative estimate as some Mg-suite melts may have assimilated crust in producing more Mg-suite material^11,33^.

**Table 1:** Model input parameters and resulting melt volume, temporal, and spatial data

	Model input			Melt Vol. (% crust)	FWHM (My)	Magmatic timing (My)	Farthest neighbor (⨴) + Exposure prop. (⨵) = ⨷					
Model	IBC (km)	Ƞ contrast	Ref. Ƞ (Pa s)				2%	3%	4%	5%	6%	7%
**Run 1**	30	10^-1^	5 × 10^20^	8.2	69.2	155.9	⨴	⨴	⨷	⨴	⨴	
**Run 2**	30	10^-2^	10^21^	5.6	37.1	92.4	⨴	⨷	⨴			
**Run 3**	30	10^-2^	5 × 10^20^	8.4	10.5	37.1	⨴	⨴	⨷	⨴	⨴	
**Run 4**	30	10^-3^	10^21^	10.0	10.6	35.6	⨴	⨷	⨴	⨴	⨴	
**Run 5**	30	10^-3^	5 × 10^20^	6.9	2.5	14.7	⨴	⨴	⨷	⨴	⨴	
**Run 6**	50	10^−2^	10^21^	13.2	20.2	55.6	⨴	⨴	⨷	⨴	⨴	⨴
**Run 7**	50	10^-2^	5 × 10^20^	17.0	7.8	25.3	⨴	⨴	⨷	⨷	⨴	⨴
**Run 8**	50	10^−3^	10^21^	15.3	7.1	23.4	⨴	⨴	⨷	⨷	⨴	⨴
**Run 9**	50	10^-3^	5 × 10^20^	17.2	3.5	12.1	⨴	⨴	⨷	⨷	⨴	⨴
**Run 10**	100	10^−4^	10^21^	25.6	3.1	6.4	⨴	⨷	⨷	⨷	⨷	⨷
**Run 11**	150	10^−4^	10^21^	18.3	1.8	3.9	⨴		⨵	⨵	⨵	⨵

Melt volume reported in vol.% of the total lunar crust. FWHM = full width half max of melt production (My), defined herein as magmatic duration.

Magmatic timing = time to 50% cumulative melt volume. Farthest neighbor = average distance between each Mg-suite detection with successful models indicated by left-half circle with “x”.

Exposure Prop. = exposure proportion of Mg-suite identifications with successful models indicated by right-half circle with “x”.

Spatial constraints of farthest neighbor and exposure proportion are evaluated within the range of Melt Detection Threshold given between 2 and 7%, with successful parameter combinations signified by an “x” inside a full circle.

### Duration and timing of lower mantle melting during cumulate overturn

Most all Runs (3–11) co-satisfy Constraints 2 and 3 by producing magmatic durations < 50 Myrs and magmatic timing within 59 Myrs of time zero (Table 1). We find that both magmatic duration (full width at half maximum of peak melt production) and magmatic timing (time measured from the onset of the model to 50% cumulative melt volume) decrease with increasing viscosity contrast (supplementary Figs. S1, S2). This is because a low viscosity contrast slows IBC downwelling and the responsive upwelling of the underlying mantle. Because the buoyant lower mantle becomes gravitationally stable during CMO relative to its initial state underlying denser cumulates^3,38^, the duration of decompression melting is finite in the absence of sustained mantle convection. At a given IBC thickness and viscosity contrast, cases with a lower mantle reference viscosity resulted in shorter magmatic durations and quicker magmatic timing relative to cases using higher mantle reference viscosity (Fig. 2b, c).

Ascent rates determined for lunar primary melts and time scales estimated for melt extraction in regions of upwelling mantle do not significantly change our results for magmatic duration or timing that are on the order of ~1–10 s of millions of years (Table 1). The Mg-suite melts must have intruded the crust in a near-primary state to explain their forsteritic olivine^10,11^, and rapid ascent rates of ~10 m s^-1^ have been determined for other primary lunar mantle-derived magmas^53^. Further, rapid separation of partial melts from their source (< 40 years) is estimated for regions of upwelling mantle^49^.

### Spatial analysis of the responsive upwelling lower mantle

**Fig. 4:**
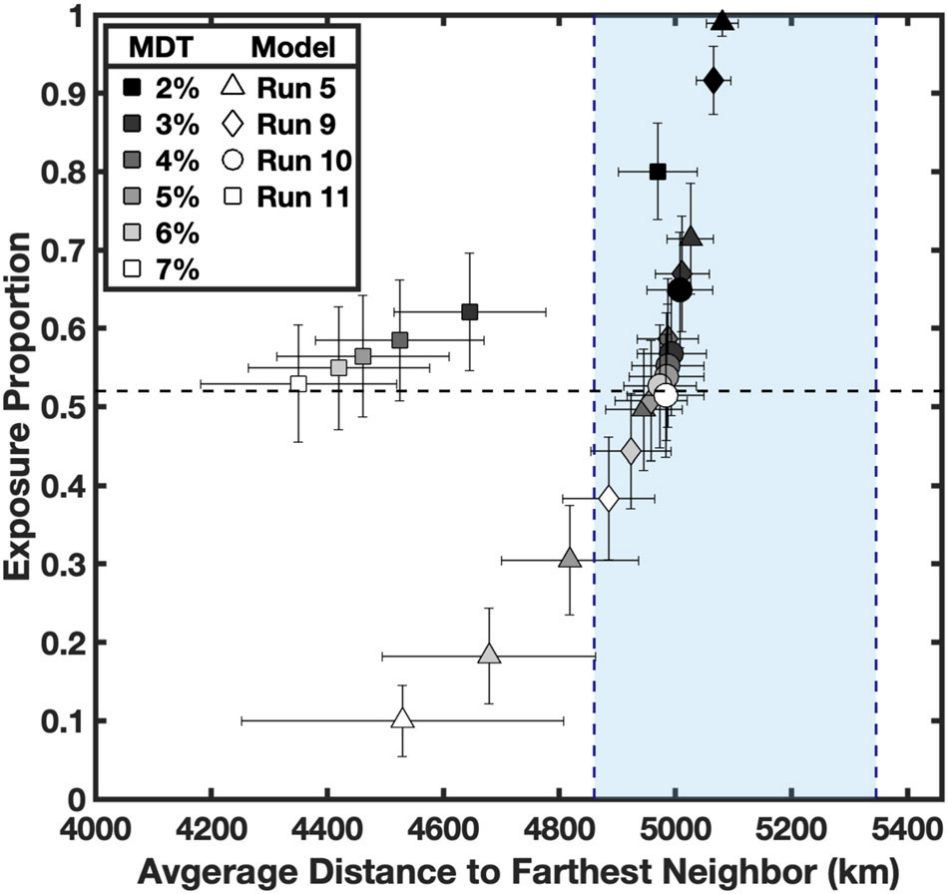
Spatial correlations of lower mantle melting induced by cumulate mantle overturn. Exposure proportion vs. average distance to farthest neighbor. The observed exposure and distance constraints of Mg-suite detections are plotted as a horizontal dashed line and blue-shaded region, respectively. Data determined from our synthetic crater modeling including 2σ standard deviation following 1000 iterations. Symbols are the same as Fig. 2, but now filled with gray scale representing each melt detection threshold considered (MDT = 2–7%). All Runs 1–10, and apart from Run 11, are capable of successfully co-satisfying the exposure and distance constraints when considering the range of MDT explored here.

In general, CMO induces widespread melting of the upwelling lower mantle matching the spatial Constraints 4 and 5 (Fig. 3, Supplementary Fig. S3). Increasing the MDT acts to decrease both the exposure proportion and farthest neighbor distance (Fig. 4). In general, most all models can simultaneously explain the observed distance and exposure constraints of Mg-suite at low to moderate degrees of partial melting (MDT = 3–5%, Table 1). Runs 10 and 11 with their thick IBC layers and high viscosity contrast are end-member scenarios that work to maximize melt volume and quicken magmatic timing within the range of possible parameter combinations defined above (Table 1). We show that despite this favorable parameter combination, Run 11 was the only model with a focused degree-1 upwelling and consequently failed to simultaneously satisfy the farthest neighbor and exposure proportion over the entire range of MDT considered.

### On the abundance, timing, and distribution of Mg-suite magmatism

**Fig. 5:**
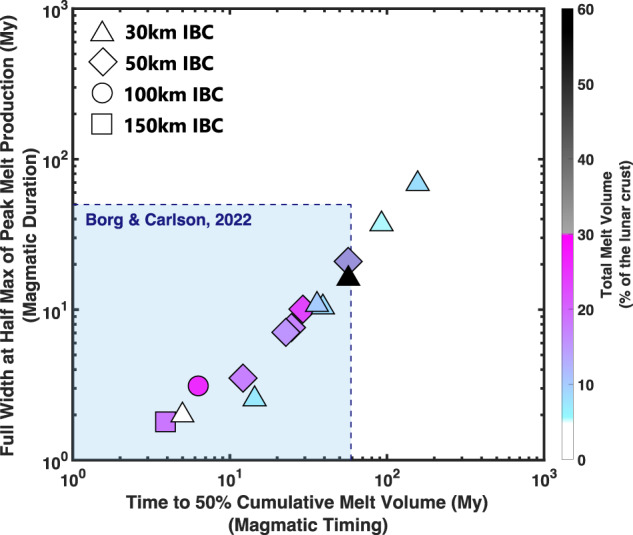
Temporal and melt volume correlations of lower mantle melting induced by cumulate mantle overturn. FWHM vs. time to 50% cumulative melt volume. Symbols are the same from Fig. 2, but now filled with the associated color scale for total melt volume (reported in vol. % of the total lunar crust). Geochronological constraints^8,30^ indicate a relatively short magmatic duration and rapid magmatic timing for Mg-suite petrogenesis (blue-shaded region). Model data shows that magmatic timing and magmatic duration are positively correlated phenomena during cumulate mantle overturn. Results indicate that partial melting of the KREEP-poor lower mantle induced by cumulate overturn can simultaneously satisfy the onset, duration, and abundance of Mg-suite magmatism.

We first emphasize that our model of CMO does not require KREEP to explain the abundance, timing, and distribution of Mg-suite rocks. Previous work^12^ has criticized the limited extent of KREEP-poor decompression melting during CMO as a shortcoming for Mg-suite petrogenesis. However, all Runs 1–11 modeled here generated melt volumes proportional to ~6–26 vol.% of the lunar crust (Figs. 2, 5). Constrained by geologically realistic initial conditions and dynamical parameters informed by experiment, our modeling demonstrates that the modest fraction of Mg-suite within the lunar crust (~6–30 vol.%) is well explained by CMO-induced decompression melting of the KREEP-poor lower mantle. Given the positive correlation between IBC thickness and Mg-suite melt abundance identified by our modeling (Fig. 2), it is also possible that incomplete participation of IBC during overturn^39,43^ limited lower mantle melting and contributed to the modest abundance of Mg-suite material observed. We therefore suggest that KREEP is not necessary for the initiation of secondary crust building on the Moon, although it may have contributed to the petrogenesis of a subset of Mg-suite samples or other episodes of lunar basaltic volcanism^12,14,15^. The incorporation of KREEP-like geochemical signatures via magma-wallrock interactions or magma mixing has been proposed as a potential secondary mechanism during Mg-suite petrogenesis^7,8,10,11,16^. Our model is thus inclusive to the observation of both KREEP-poor and KREEP-bearing Mg-suite rock types in the meteorite and sample collection when considering KREEP as a possible contaminant during, instead of the driver of, Mg-suite magmatism.

**Fig. 6:**
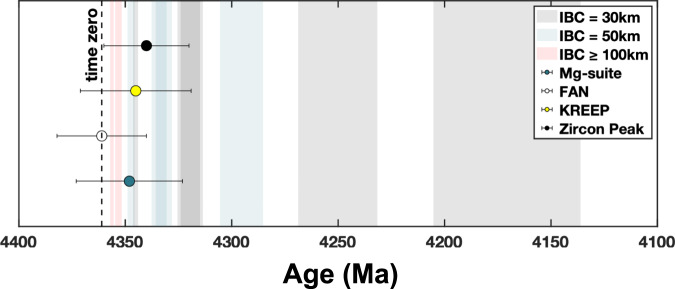
Summary of ages for primary LMO products relative to secondary Mg-suite magmatism and magmatic timing and duration results from our modeling. Legend provided for model data (colored bars) and geochronological data (filled-circles with error bars)^8,30^. Left-most edge of colored bars represent the onset of Mg-suite magmatism (time to 50% cumulative melt volume) relative to its duration (defined by the width of a given bar). Assigning a time zero of our model consistent with primary FAN closure (4361 Ma) suggests that CMO-induced decompression melting of the KREEP-poor lower mantle naturally explains the rapid transition from primary to secondary crust building on the Moon in addition to a limited duration of Mg-suite magmatism.

Importantly, our modeling shows that the CMO process alone can reconcile the concordant formation ages between the primary flotation crust (FAN) and secondary Mg-suite (Figs. 5, 6). Chronological constraints used in this study are derived from concordant dating of Mg-suite rocks and concordant ages of FAN^8,30^. Collectively, these data indicate a relatively short magmatic duration for Mg-suite and quick magmatic timing relative to FAN closure. A major result is that our modeling naturally aligns with these two chronological constraints, as we demonstrate that magmatic duration and magmatic timing are positively correlated phenomena for CMO-induced magmatism (Fig. 5). In this way, the short interval between FAN and Mg-suite formation and the brief duration of Mg-suite magmatism revealed by geochronology are naturally explained by CMO (Fig. 6). If instead a large amount of radiogenic KREEP was incorporated into the Mg-suite source, this prolonged supply of heating should extend the magmatic duration of Mg-suite beyond current observations, further questioning the role of KREEP in driving short-lived Mg-suite magmatism.

Implicit in the near-concordant dates of FAN is that the LMO solidified near 4361 Ma^8,26,28,30^. Other chronological approaches suggest LMO solidification occurred earlier, and perhaps as early as 4510 Ma^54,55^. If the earlier LMO solidification dates are accurate, this would require CMO-induced Mg-suite magmatic timing on the order of ~100–150 Myrs. Runs 1 and 2 with 30 km thick IBC and low viscosity contrast (10^-1^–10^-2^) produce magmatism on this timescale (Figs. 2c, 6). However, the magmatic duration of Run 1 extends beyond the current constraint of 50 Myrs (Fig. 2b). In this context, we stress that thin IBC layers should be most enriched in ilmenite^37^. Because ilmenite is rheologically weak, a thin, ilmenite-rich IBC layer with low viscosity contrast is not a geologically or experimentally supported parameter combination^41^. Our higher viscosity contrast (10^-3^–10^-4^) models are therefore better aligned with rheological expectations and uniformly produce magmatic timing in < 59 Myrs and magmatic durations < 50 Myrs. Reconciling an older FAN formation age (~4.5 Ga) with Mg-suite petrogenesis by IBC-driven CMO may require future revisions to lunar chronology and the rheology of LMO cumulates. Whereas we show that near-contemporaneous primary and secondary crust building is entirely consistent with current geochronological and rheological constraints (Fig. 5).

Another major finding from our dynamical modeling is that the CMO process commonly leads to widespread upwelling and partial melting of the KREEP-poor lower mantle (Fig. 3, Supplementary Fig. S3). This is important because we show that widespread upwelling and partial melting of the lower mantle in response to CMO provides explanation for the global detections of early secondary crust in the remote sensing database (Fig. 1). Our synthetic crater modeling (Fig. 4) specifically indicates that the melt distribution from CMO with degree > 1 upwelling can co-satisfy the exposure and distance constraints at low to moderate degrees of partial melting where MDT = 3–5% (Table 1, Fig. 4). Our results therefore eliminate degree 1 lower mantle upwelling as a viable scenario because the focused and hemispheric melt distribution of Run 11 violates the coupled exposure proportion and distance constraints (Fig. 4). Consequently, our results do not support thick IBC layers = 150 km with a high viscosity contrast. Regardless, our study underscores the significance of integrating orbital remote sensing of early secondary crust to further constrain the extent and styles of initial mantle convection on the Moon.

Finally, our model considers mantle overturn driven by the dense IBC layer within a fully solidified Moon. Next, we discuss our results within the context of two alternative scenarios below: silicate overturn initiating prior to complete LMO solidification, and overturn induced by the giant South Pole-Aitken basin forming impact.

### Implications concerning a long-lived residual magma ocean

The first ~80% of LMO solidification is likely rapid^38^, whereas the presence of an insulating FAN lid can extend the duration of the final ~20% of LMO crystallization up to ~200 Myrs^38,55,56^. This extended duration of LMO solidification could exceed the time to initiate silicate-driven mantle overturn^38^ unless a rigid mantle viscosity is assumed (10^22 ^Pa s)^55^ or rapid compaction of the cumulate pile led to a metastable mantle stratigraphy^24,57^. Silicate-driven mantle convection is thus possible in a long-lived, partially solidified magma ocean^55,56^, and could result in syn-FAN decompression melting. If so, silicate overturn generally works in favor of reconciling a contemporaneous relationship between primary FAN and secondary Mg-suite. Nevertheless, petrologic and geochronologic context requires that FAN production preceded secondary magmatic intrusions.

Here we note that LMO models^38–40,58–60^ predict formation of the high-density IBC layer after FAN production and, perhaps more importantly, prior to both urKREEP and complete LMO solidification. This is important because the formation of IBC reduces overturn initiation timescales to thousands of years^38^. Our results of IBC-driven overturn therefore remain valid considering long-lived residual magma oceans since the time zero of our model is predicated on the isotopic closure ages of FAN and not the complete solidification age of the LMO (Fig. 6). The hot and positively buoyant Mg-suite melts generated by decompression melting (1 bar liquidus ~1563 °C, liquidus density ~2789 kg m^-3^)^11,34^ are thus capable of ascending through the cool (~1000-1150 °C) and relatively dense (~2893–3161 kg m^-3^) syn-FAN residual magma ocean^58,59^. Such a scenario could account for both Mg-suite primary melts acquiring evolved trace element characteristics from the residual magma ocean in addition to buoyancy forces predominantly controlling Mg-suite melt transport^34^. Regardless, our results imply that an IBC layer formed within millions to tens of millions of years of FAN closure to satisfy the geochronologic constraints of Mg-suite magmatism.

### Initiation of overturn by the South Pole-Aitken impact?

An alternative hypothesis to IBC-driven overturn is that the South Pole-Aitken (SPA) impact triggered overturn of a metastable mantle stratigraphy^61–65^, ultimately resulting in the observed geochemical asymmetry of the lunar surface^61,62,66^ and potentially leading to Mg-suite production. In this scenario, widespread mantle convection like our modeling shows can be rapidly (within hours) induced by thermal anomalies from the SPA impact^61^. If secondary crust building was initiated during this SPA-induced stage of early mantle convection, geochronology then requires that the SPA impact be coincident with primary crust formation at ~4361 Ma. A minimum age of ~4.3 Ga has been inferred for SPA based on a reexamination of the areal density of impact craters using Gravity Recovery and Interior Laboratory data^67^, and is thus consistent with the hypothesis above. However, this scenario ultimately remains untestable by radiometric dating methods in the absence of samples returned from SPA.

### Implications for the initial temperature profile of the lunar mantle

We now discuss results from a set of models that test cooler and hotter initial temperature profiles for the LMO cumulates compared to that considered above. It is clear from our spatial analysis and range of melt detection threshold that CMO, with the exception of Run 11 and its degree 1 upwelling, is capable of explaining the global distribution of Mg-suite observed by orbital spacecraft regardless of timing and melt volume constraints (Table 1). Our focus here therefore turns to magmatic timing, magmatic duration, and total melt volume as potential discriminators for testing the pre-overturn initial temperature of the lunar mantle.

If the LMO cumulate layers compacted rapidly to form a metastable mantle stratigraphy^24,57^, then the lower mantle may have cooled through conduction prior to overturning. In this case, the temperature profile of the Mg-suite source could be cooler than what has been thus far considered. To test our model in this scenario, we report Run 3C (Supplementary Table S3) which is identical to Run 3 but considers an initial conductive temperature profile in the lower mantle relative to the peridotite solidus (supplementary Fig. S7). Run 3C was ultimately terminated because it became apparent that it would not satisfy the natural observations having not reached its peak melt production rate after 114 Myrs in addition to producing very little lower mantle melting over this timeframe (~0.04 vol.% of the lunar crust). Following, we lowered the mantle reference viscosity (5 x 10^19^ Pa s) to promote quicker magmatic timing and to fully quantify the overturn process in this scenario (Run 3C_i). Despite satisfying the geochronological constraints with this low reference viscosity (Supplementary Table S3), upwelling of the cool lower mantle in Run 3C_i again resulted in low total melt volume (~0.03 vol.% of the lunar crust).

Runs 1H and 6H are identical to Runs 1 and 6, respectively, but test a hotter initial temperature profile. Pure fractional crystallization of the LMO should result in each mantle horizon having a unique and compositionally dependent solidus and liquidus in the absence of cumulate mixing. To account for this we assume that mantle layers formed and accumulated at a temperature between the peridotite liquidus and solidus during a bottom-up fractional crystallization sequence of the LMO. The initial temperature for every cumulate horizon is calculated assuming that LMO melt fraction varies linearly between the solidus and liquidus as a function of temperature (Supplementary Fig. S8). We then account for the compositional dependency on the solidus and liquidus in our modeling by calculating new solidii and liquidii for each radial element in our model Moon. We do this by quantifying the offset between the peridotite solidus and liquidus and translate this offset to the hotter initial temperature profile at a given radial element, and then the depth-dependent offset of the peridotite solidus and liquidus is followed to shallower depths to produce 64 new and independent solidii and liquidii for melting calculations (Supplementary Fig. S8).

Because viscosity is temperature dependent, the hotter initial temperature works to decrease magmatic timing and duration, as observed in comparable runs varying only reference viscosity (e.g., Runs 2 vs. 3, 4 vs. 5). Run 6H produced magmatic duration and timing of 10 and 29 Myrs, respectively, compared to 20 and 56 Myrs for Run 6. Run 6H yielded a total melt volume equivalent to 23 vol.% of the lunar crust compared to 13 vol.% produced by Run 6. Run 1H resulted in a magmatic duration and timing of 16 and 58 Myrs, respectively (compared to 69 and 156 Myrs in Run 1). Run 1H also yielded a total melt volume equivalent to 57 vol.% of the lunar crust (compared to 8.2 vol.% in Run 1).

Our additional modeling provides new insight into the temperature profile of the lunar mantle at the onset of cumulate overturn. Within the evidence-based framework indicating a petrogenetic link between CMO and Mg-suite, the insufficient melt volumes produced by Runs 3C and 3C_i suggests that thermal conduction of the lower mantle could not have been extensive at the time of overturn. A cool pre-overturn cumulate pile is therefore not favored. Instead, hotter initial temperatures work to decrease magmatic timing and duration, consistent with constraints from geochronology (supplementary table S3). An overproduction of total melt volume, and therefore an overabundance of Mg-suite within the crust, can result however (Run 1H vs. Run 1). The melt production constraint could still be satisfied with a hot cumulate pile if the IBC viscosity contrast is minimized, reference viscosity is maximized, or if IBC layer thickness is minimized (Fig. 2). Alternatively, melt production constraints could be satisfied for a hot cumulate pile if a large fraction of melt remained trapped below the crust. Thus, a hot cumulate pile remains a viable scenario, albeit with a relatively narrow associated parameter space as constrained by our dynamical models.

### Secondary crust building on the Moon and differentiated bodies

Within the range of input parameters constrained by natural observation, experiment, and numerical simulations, our dynamical modeling identifies that widespread decompression melting of KREEP-poor primary magma ocean cumulates in response to overturn of 30–50 km thick IBC (possibly up to 100 km) can reproduce the key volume, geochronological, and spatial characteristics of the earliest secondary crust on the Moon (Figs. 3–5). Importantly, our model of origin establishes a direct link between CMO and initiation of secondary crust building, and is therefore consistent with hypotheses that Mg-suite petrogenesis was not itself driven by KREEP^7,8,10,11,16^. Instead, KREEP geochemical signatures could have been obtained via secondary processes such as magma mixing or melt-rock interactions during ascent of partial melts derived from the upwelling lower mantle. Our modeling thus remains in agreement with calculated ^147^Sm/^144^Nd and ^87^Rb/^86^Sr ratios of the Mg-suite source region^8^ which link to a source that formed coincidently with LMO differentiation as discussed above, or alternatively, a primitive and undifferentiated mantle component.

Natural observations associated with secondary crust building are best explained by mantle overturn when considering a low mantle reference viscosity (5 × 10^20^ P s) and high viscosity contrast of 10^-2^ or greater. This is because lowering the reference viscosity of the mantle serves to decrease the magmatic duration and quicken magmatic timing (Fig. 2b, c). Successful models using a high mantle viscosity (10^21^ P s) required a greater viscosity contrast with the IBC layer (Table 1) or higher initial temperatures (Supplementary Table S3). The range of reference viscosities used here is consistent with the rheology determined for dry peridotite^37^, but it is possible that water^68–73^ and trapped melt^38,74,75^ act to lower cumulate viscosity within the LMO^76^ and thus quicken magmatic timing and minimize magmatic duration during CMO. Our results therefore suggest that overturn of rheologically weaker cumulates than tested here would further support a contemporaneous relationship between primary and secondary crust building on the Moon. As a corollary, our results imply that overturn on differentiated bodies with rigid mantles or a low viscosity contrast could result in a temporal disconnect between primary and secondary crust building in addition to prolonged periods of initial secondary magmatism relative to that observed on the Moon (Fig. 6). Initial temperature profiles of the lunar mantle equivalent to or hotter than the peridotite solidus remain viable scenarios, and are consistent with estimated mantle potential temperatures (> 1600 °C) at the time of Mg-suite formation^45^. Significant thermal conduction of the lunar mantle prior to overturn, and thus a cool pre-overturn temperature profile, is not favored on the basis of insufficient secondary crust production (Supplementary Table S3). Regardless, our study highlights the importance of future sample return missions, detailed surface exploration via orbital remote sensing, and further radiometric dating toward constraining the dynamical evolution of the Moon and terrestrial planets.

We therefore conclude that CMO-induced decompression melting of KREEP-poor LMO mantle cumulates can explain the rapid transition from primary to secondary crust building on the Moon revealed by geochronology (Fig. 6). The lunar Mg-suite provides foundational evidence for the hypothesis that gravitational instabilities in magma ocean cumulate piles are major driving forces for the dynamics of early mantle convection within and initial secondary crust building on differentiated bodies^3,24,37,77–81^. Our work supports this hypothesis and implies that the influence of global-scale magma oceans remains central to planetary evolution, even after their solidification is complete.

## Methods

### Model parameters and inputs

Our 3D model of mantle overturn uses a 12 × 64 × 48 × 48 mesh based on CitcomS^82^, which gives an azimuthal resolution of 14 km. Grids are refined radially at the top and bottom boundary to resolve the thermal boundary layers and IBC layer. Comparison to modeling with finer radial resolution (7 km) demonstrates that the IBC layer is well resolved by our calculations (Supplementary Fig. S4). The core-mantle boundary, the lower-upper mantle boundary, the IBC bottom, and the IBC-crust boundaries are also defined by magma ocean modeling^38^ and are accordingly set at the nominal radii of 340 km, 1040 km, 1660 km, and 1710 km, respectively.

The evolution of the four silicate layers is solved with conservation of mass, momentum, and energy. We apply the general derivation^46^ of

1 $$F=\frac{T-{T}_{{{{{\mathrm{solidus}}}}}}}{{T}_{{{{{\mathrm{liquidus}}}}}}-{T}_{{{{{\mathrm{solidus}}}}}}}$$

where *F* is the weight fraction of melt, *T* is temperature, *T*_liquidus_ is the liquidus temperature, and *T*_solidus_ is the solidus temperature to calculate the local production of lower mantle melting using the peridotite or recalculated effective solidii and liquidii (Supplementary Figs. S5, S7, S8). We thus use Eq. (1) as a proxy for Mg-suite melt volume as hypothesized in previous work^4,8,11^. Following previous work^37^ (Supplementary Table S2) the mantle thermal Rayleigh number is set to 6 × 10^5^. Although the latent heat is applied in every case, the effect of latent heat is not sound at the temperature profile of the lower mantle because the azimuthally averaged temperature of the lower mantle is barely higher than the solidus (Supplementary Fig. S6). The thermal conductivity of the crust^37^ is set to 4 W m^-1^ K^-1^. As a test, we performed an additional test of Run 1 using a lower conductivity of 2 W m^-1^ K^-1^ for the crust (Run 1a) but did not find any significant changes to our results (supplementary table S3). Our initial thermal condition for Runs 1–11 and Run 1a considers a peridotite solidus and has a 90-km top thermal boundary layer.

Our model assumes 50% of all heat producing elements (U, Th, and K) are concentrated in the IBC layer^3^, while the remaining 50% are evenly distributed throughout the lunar crust and mantle^83–86^. This distribution of heat producing elements is based on the IBC forming in the final stages of magma ocean crystallization^38,58–60^ and evolves dynamically afterward. The heat generation rate of these heat producing elements is calculated based on the bulk U and Th abundances of the Moon. The bulk U and Th abundances of the present day are taken as 25.7 and 102.8 ppb (Th/U = 4), respectively^87^. The Moon is highly depleted of the volatile element K^88–90^, and we apply a K/Th ratio of 2500^66^. A major finding of this work is that the origin of Mg-suite can be explained by decompression melting of the lower mantle and thus, independently from the distribution of KREEP.

Numerical and experimental simulations of LMO crystallization predict thin IBC layers (≤ 50 km) based on mass balance and phase equilibria, and thicker IBC layering up to 150 km is possible when considering dynamic redistribution of IBC diapirs during the LMO solidification process^3,37^. Following previous work^37^, we therefore treat the initial thickness of the ilmenite-bearing cumulate (IBC) layer as a free parameter by modeling thicknesses of 30, 50, 100, and 150 km to explore the effects of IBC thickness on the dynamic return flow patterns and decompression melting of the lower mantle (Mg-suite source).

The viscosity contrast between the lunar mantle and IBC plays a key role in determining the dynamics of CMO^41–43^. Viscosity is both temperature and compositionally dependent and we explore the range of IBC viscosities both constrained by experiment^41^ and defined in previous modeling^37^. Following previous work^37^, we vary the reference viscosity of the lunar mantle with the approximated rheology of peridotite, which can range from 5 × 10^20^–10^21 ^Pa s (Table 1). Ilmenite is rheologically weak, and the viscosity of pure ilmenite is up to 4 orders of magnitude lower than that of dry peridotite^37^. The viscosity of the IBC layer itself is complicated by the ilmenite fraction, IBC thickness (which is dependent on LMO composition), water content, and melt fraction^37^. It is for these reasons that we treat the viscosity contrast between the IBC and underlying mantle as a free parameter varying from 10^-1^–10^-4^. Considering the IBC thicknesses explored here, the possible ilmenite fraction of the IBC layer is estimated to be ~1.5–11.5 vol.%, corresponding to a viscosity contrast ≥ 3 orders of magnitude^37,41^. We also present results from end-member cases such as thin IBC layers paired with a low viscosity contrast (e.g., Run 1) and thick IBC layers with the viscosity contrast considering pure ilmenite (e.g., Runs 10, 11) to explore a range of possible physical combinations.

### Supplementary information

Supplementary Information

## Data Availability

Processed data generated in this study are included in this published article (and its supplementary information files).

## Code availability

CitcomS is an open-source software available at Computational Infrastructure for Geodynamics (https://geodynamics.org/cig/software/citcoms/).
