# Supplementary material for: Rapid transition from primary to secondary crust building on the Moon explained by mantle overturn
Source: Nat Commun. 2023 Aug 17;14:5002. doi: 10.1038/s41467-023-40751-7 (PMC10435462; doi:10.1038/s41467-023-40751-7)
Supplement: Supplementary file 1 — Supplementary Information [file 41467_2023_40751_MOESM1_ESM.pdf]

Rapid transition from primary to secondary crust building on the Moon explained by mantle overturn.

by
Tabb C. Prissel[1*]
Nan Zhang[2,3*]
Colin R.M. Jackson[4]
Haoyuan Li[5]

[1]NASA Johnson Space Center
Astromaterials Research & Exploration Science Division
2101 NASA Parkway, MailCode XI3
Houston, TX 77058, United States

[2]Key Laboratory of Orogenic Belts and Crustal Evolution
School of Earth and Space Sciences
Peking University
Beijing, China 100871

[3]School of Earth and Planetary Sciences
Curtin University
GPO Box U1987, WA 6845, Australia

[4]Department of Earth and Environmental Sciences
Tulane University
6823 St. Charles Avenue
New Orleans, LA 70118-5698, United States

[5]Department of Earth and Planetary Sciences
University of California, Davis
One Shields Avenue
Davis, CA 95616, United States

*Corresponding authors: tabb.c.prissel@nasa.gov; nan_zhang@pku.edu.cn

Supplementary Information

Contents of this document include:

- **Supplementary Fig. S1. Melt production vs. time.**
- **Supplementary Fig. S2. Total melt volume vs. time.**
- **Supplementary Fig. S3. Morphology and melting of upwelling lower mantle in response to cumulate mantle overturn.**
- **Supplementary Fig. S4. Resolution of the IBC layer.**
- **Supplementary Fig. S5. Evolution of temperature profiles in the mantle.**
- **Supplementary Fig. S6. Effect of Latent Heat.**
- **Supplementary Fig. S7. Initial temperature profile of conductive mantle overturn scenario.**
- **Supplementary Fig. S8. Initial temperature profile of hot mantle overturn scenario and new solidii and liquidii.**
- **Supplementary Table S1. Temporal, volume, and spatial data from dynamical models evaluated with respect to defined criteria.**
- **Supplementary Table S2. Model input parameters.**
- **Supplementary Table S3. Additional Tests of Model Input Parameters Relative to Runs 1 - 11 in Table 1.**

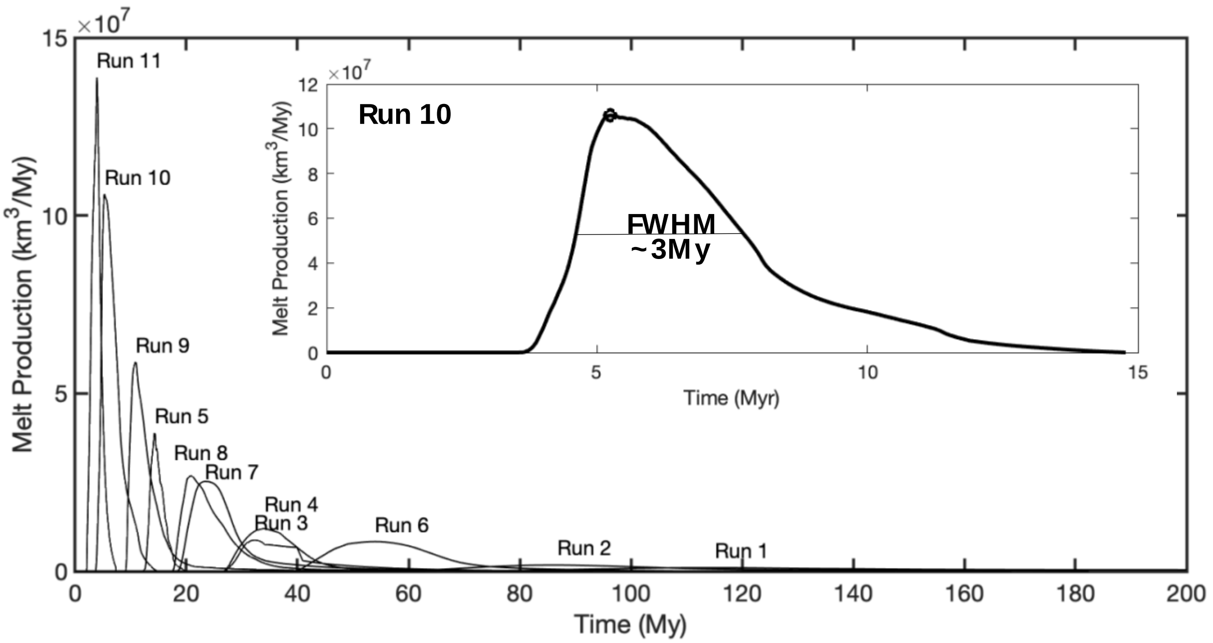

Supplementary Fig. S1. Melt production vs. time. The rate of melt production (km^3/Myrs) for each Run is plotted as a function of time. Inset demonstrates quantification of our “magmatic duration” for Run 10 by measuring the full width at half maximum of the peak melt production curve.

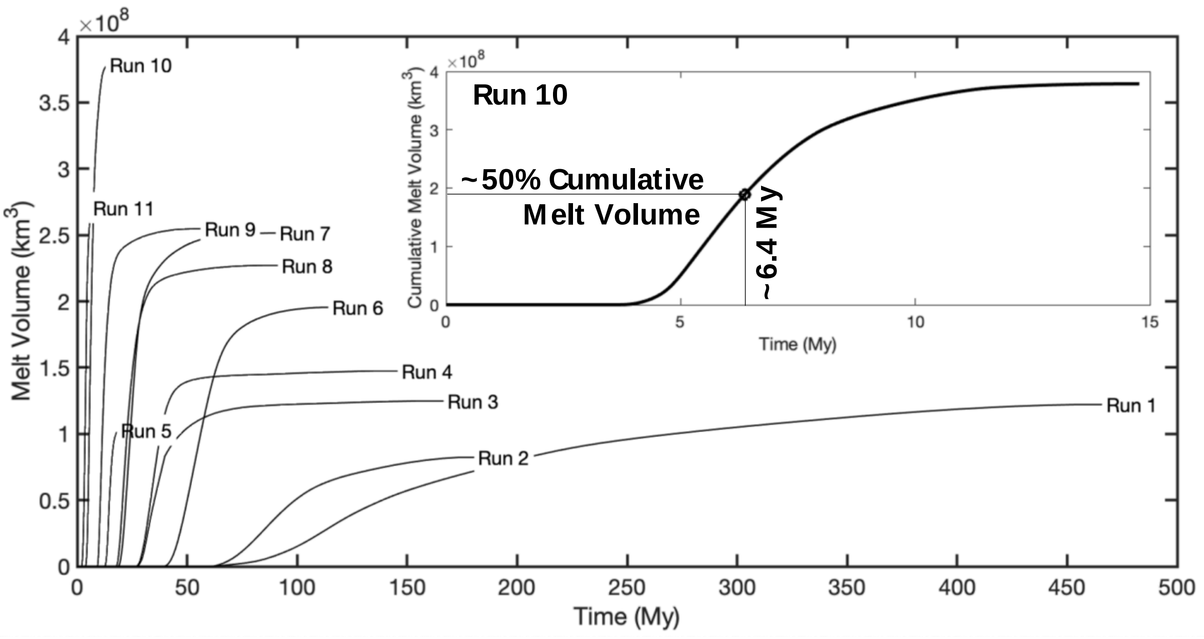

Supplementary Fig. S2. Total melt volume vs. time. The cumulative melt volume for each Run is plotted as a function of time. Inset demonstrates quantification of our "magmatic timing" for Run 10 by quantifying the time step most closely associated with 50% cumulative melt volume.

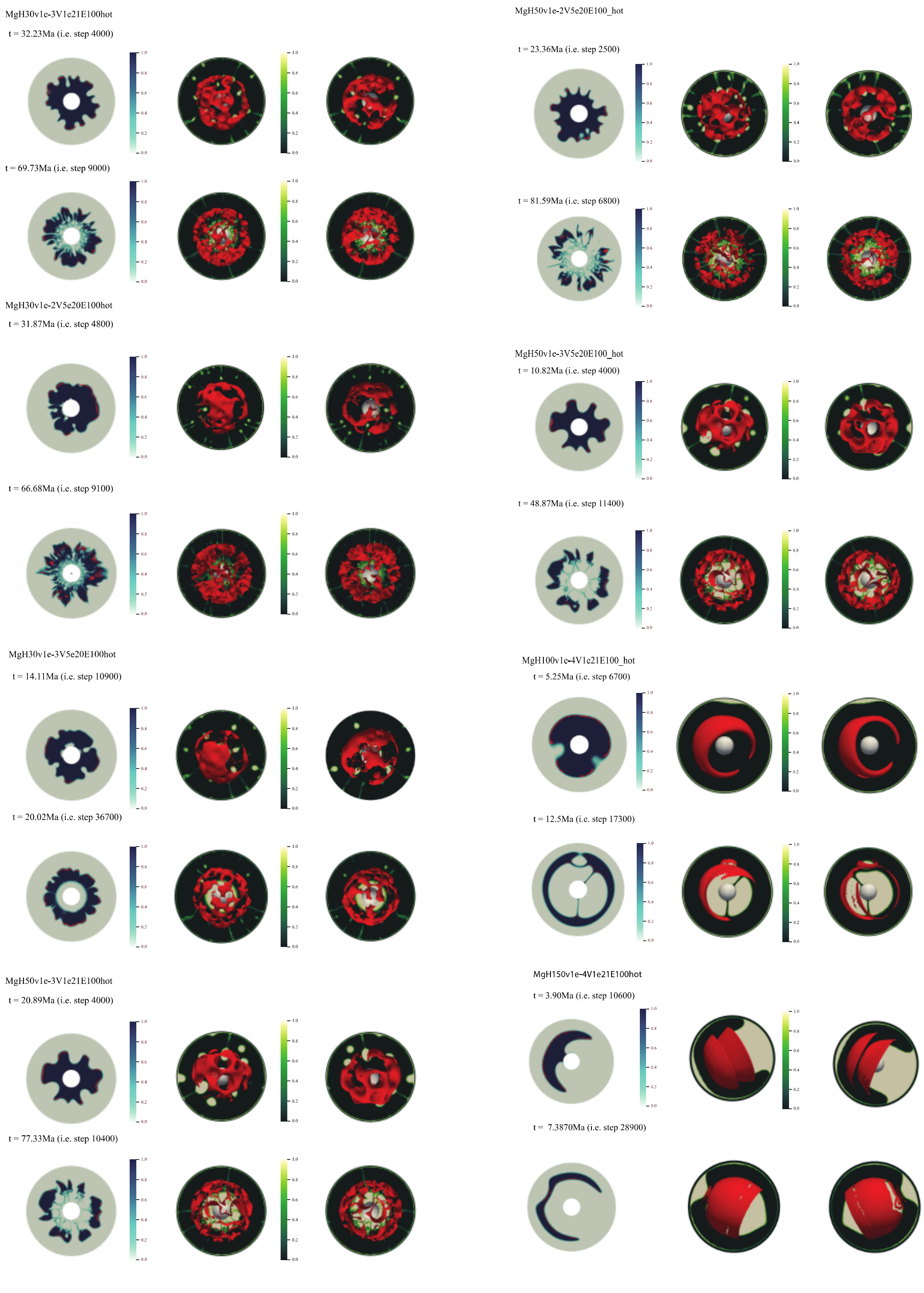

Supplementary Fig. S3. Morphology and melting of upwelling lower mantle in response to cumulate mantle overturn. Additional cases and perspectives highlighted in Fig. 2. Presented are snap shots near peak melt production and near the end of each mode run. Left: isolating the 2-D morphology of upwelling layer-0 (layer-0 color scale provided) and associated regions of melting (red). Right: visualization of the 3-D melt surface of layer-0 (red) overlain on top of a 2-D slice of the downwelling IBC (IBC color scale provided).

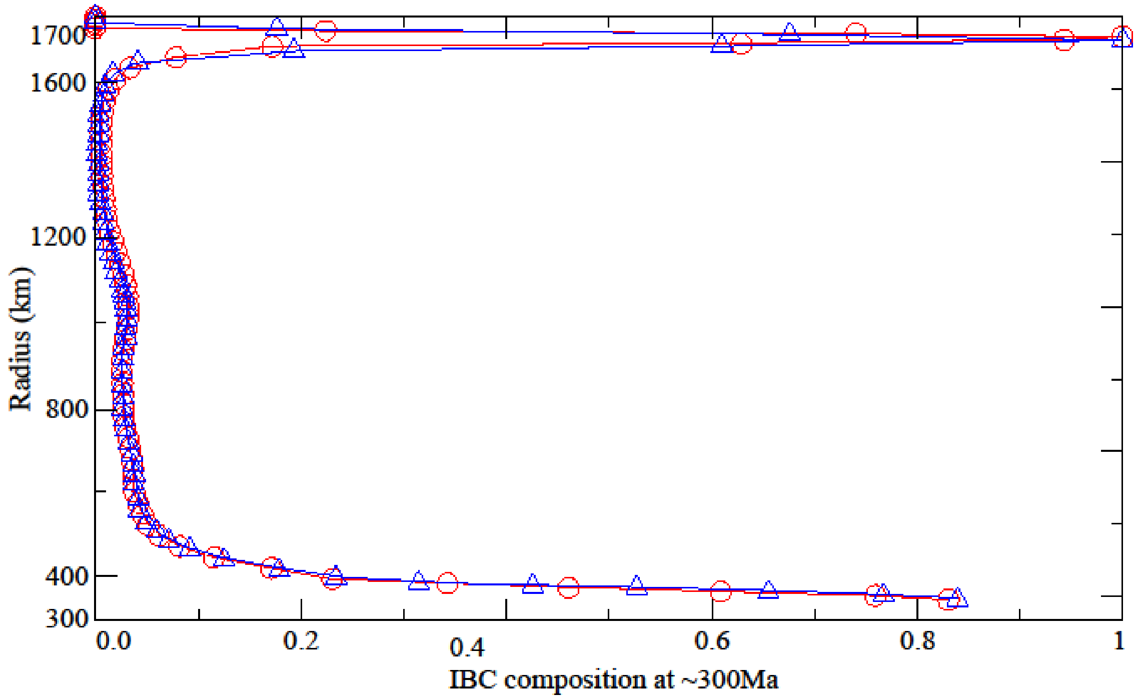

Supplementary Fig. S4. Resolution of the IBC layer. Our modeling included a top boundary radial resolution of 22 km (blue triangles). We compare this to a higher resolution case with 7 km radial resolution (red circles) to demonstrate that our calculations resolve the IBC layer well.

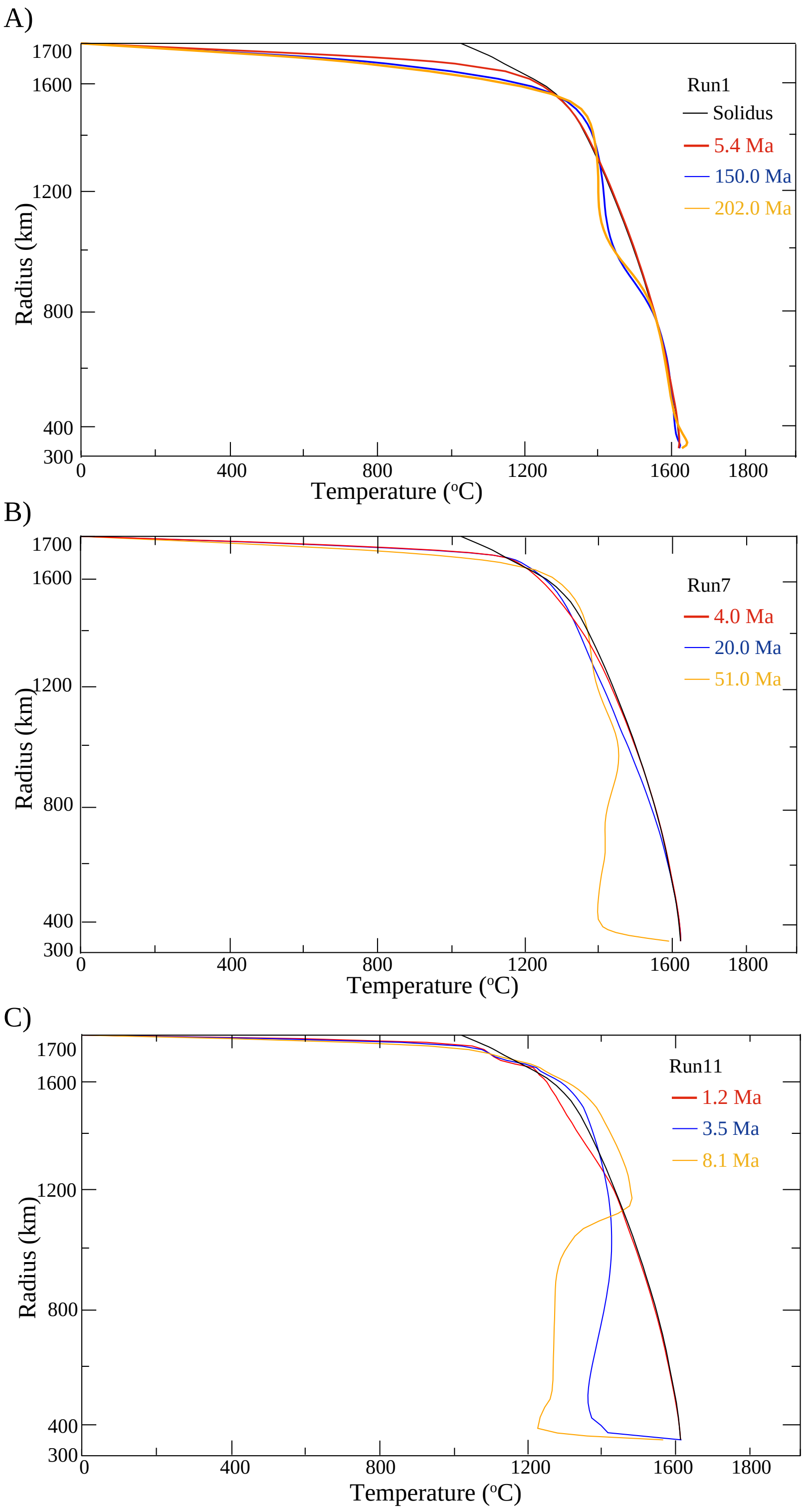

Supplementary Fig. S5. Evolution of temperature profiles in the mantle. Radius vs. temperature and evolution of temperature profiles for Runs 1 (A), 7 (B), and 11 (C). The solidus is plotted as the black curve in each. Snapshots of temperature profiles are shown by red, blue, and yellow curves with specific times provided in the respective legend.

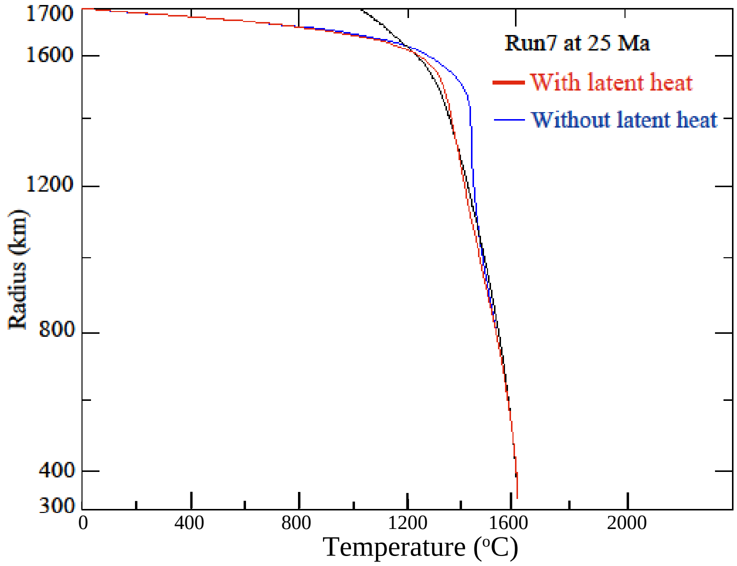

Supplementary Fig. S6. Effect of Latent Heat. Radius vs. temperature as in supplementary figure S5 with solidus (black curve) and azimuthally averaged temperature profiles considering latent heat (red curve) and without latent heat (blue curve) at 25 Myrs for Run 7. The effect of latent heat is not sound at the temperature profile of the lower mantle because the azimuthally averaged temperature of the lower mantle is barely higher than the solidus.

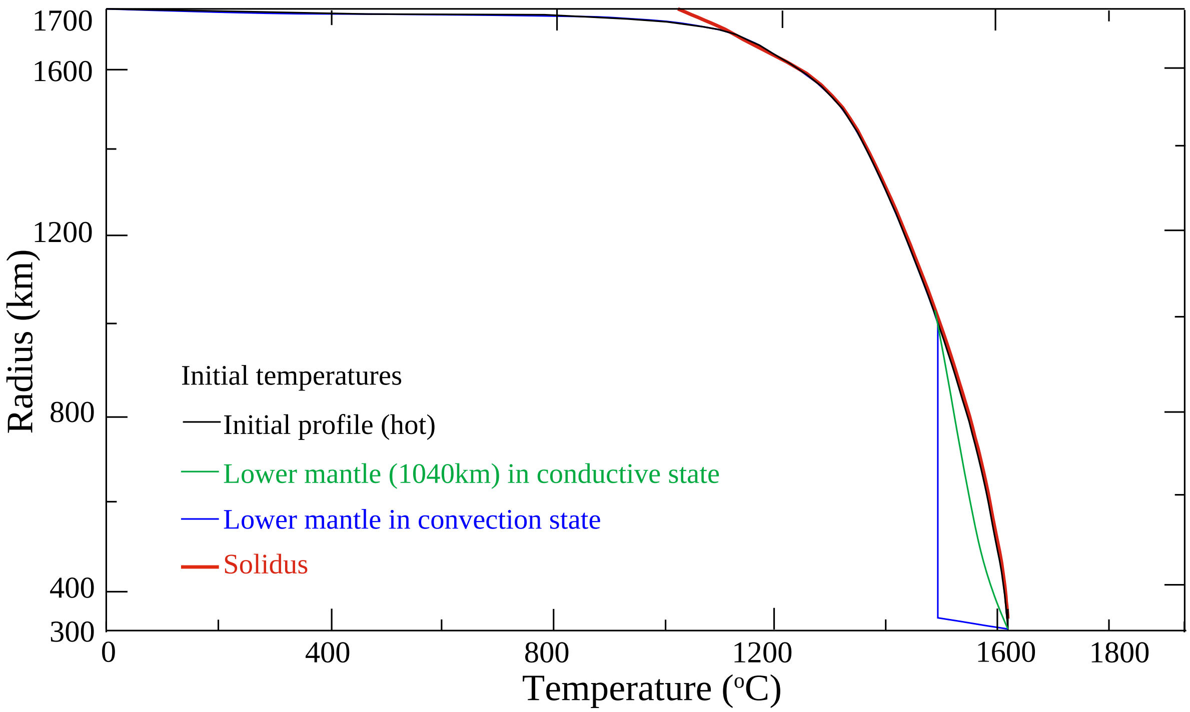

Supplementary Fig. S7. Initial temperature profile of conductive mantle overturn scenario. Radius vs. temperature as in supplementary figure S5 with solidus (red curve), initial temperature profile (black curve), and lower mantle temperature profile considering thermal conduction (green curve) and that in a convective state (blue curve).

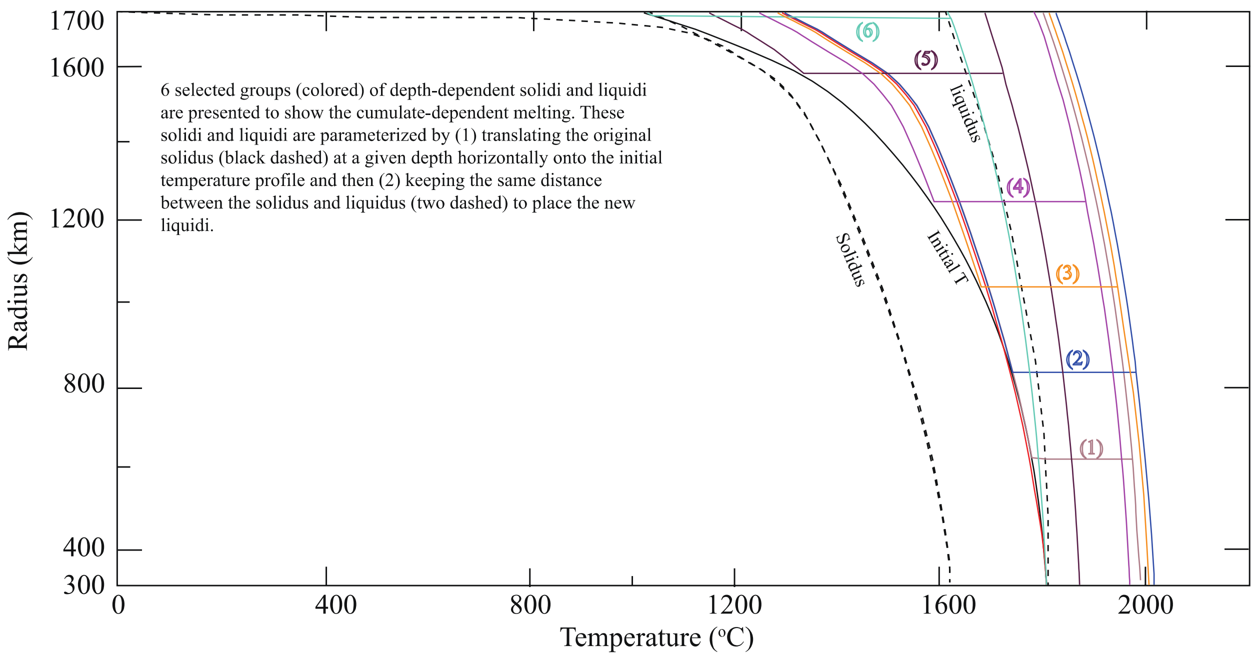

Supplementary Fig. S8. Initial temperature profile of hot mantle overturn scenario and new solidii and liquidii. Radius vs. temperature as in supplementary figure S7 with peridotite solidus and liquidus labeled and given by dashed curves, the hot initial temperature profile also labeled and given by the solid black curve. Six examples are shown for calculating new and compositionally dependent solidii and liquidii relative to the hot initial temperature profile.

Supplementary Table S1. Temporal, volume, and spatial data from dynamical models evaluated with respect to defined criteria.

	**Exposure Proportion (detections per crater examined) [0.52]**						**Farthest Neighbor (km) [5103 ± 243 km]**					
**Model**	**2%**	**3%**	**4%**	**5%**	**6%**	**7%**	**2%**	**3%**	**4%**	**5%**	**6%**	**7%**
**Run 1**	0.97 (0.03)	0.64 (0.07)	0.48 (0.08)	0.35 (0.07)	0.27 (0.07)	0.20 (0.06)	5078 (28)	5007 (51)	4940 (70)	4869 (105)	4790 (119)	4692 (164)
**Run 2**	0.93 (0.04)	0.56 (0.08)	0.36 (0.08)	0.23 (0.07)	0.14 (0.05)	0.09 (0.05)	5071 (29)	4953 (62)	4832 (112)	4658 (164)	4490 (244)	4257 (389)
**Run 3**	0.98 (0.02)	0.61 (0.08)	0.46 (0.08)	0.36 (0.07)	0.27 (0.07)	0.20 (0.06)	5080 (28)	4990 (50)	4928 (72)	4844 (104)	4766 (135)	4675 (168)
**Run 4**	0.93 (0.04)	0.54 (0.08)	0.42 (0.08)	0.34 (0.08)	0.29 (0.07)	0.24 (0.07)	5075 (28)	4985 (56)	4937 (75)	4907 (92)	4861 (99)	4847 (111)
**Run 5**	0.99 (0.01)	0.71 (0.07)	0.50 (0.08)	0.31 (0.07)	0.18 (0.06)	0.10 (0.05)	5081 (27)	5025 (40)	4945 (66)	4818 (118)	4679 (183)	4530 (277)
**Run 6**	0.93 (0.04)	0.60 (0.07)	0.49 (0.07)	0.40 (0.08)	0.35 (0.07)	0.30 (0.07)	5072 (29)	4978 (54)	4915 (71)	4850 (84)	4805 (100)	4759 (120)
**Run 7**	0.93 (0.04)	0.61 (0.07)	0.51 (0.08)	0.44 (0.08)	0.39 (0.08)	0.35 (0.08)	5071 (30)	4977 (53)	4931 (70)	4882 (85)	4840 (96)	4814 (101)
**Run 8**	0.91 (0.04)	0.66 (0.08)	0.56 (0.07)	0.47 (0.08)	0.40 (0.08)	0.34 (0.07)	5068 (30)	5010 (48)	4975 (57)	4937 (65)	4891 (80)	4848 (90)
**Run 9**	0.92 (0.04)	0.67 (0.07)	0.59 (0.08)	0.51 (0.08)	0.44 (0.07)	0.38 (0.08)	5066 (30)	5011 (47)	4987 (53)	4958 (62)	4924 (70)	4885 (80)
**Run 10**	0.65 (0.07)	0.57 (0.08)	0.55 (0.08)	0.54 (0.08)	0.53 (0.08)	0.51 (0.08)	5008 (57)	4993 (60)	4987 (63)	4984 (65)	4973 (63)	4983 (67)
**Run 11**	0.8 (0.06)	0.62 (0.08)	0.58 (0.08)	0.56 (0.08)	0.55 (0.08)	0.53 (0.07)	4969 (68)	4646 (130)	4525 (145)	4461 (149)	4420 (156)	4350 (169)

FWHM = full width half max of melt production (My); CMO-Mg-suite = the time to reach 50% total melt volume; Vol.% = total volume of melt reported as % of total crust volume

Exposure Proportion = proportion of positive Mg-suite identifications per crater examined; Farthest Neighbor = average distance between each Mg-suite detection and its farthest neighbor.

Constraints defined for each criterion are displayed in brackets [ ]; 2σ standard deviation reported in parentheses. Melt Detection Threshold (MDT) was varied between 2 - 7%.

Supplementary Table S2. Model input parameters.

Parameters	Value
Moon radius, R	1740 km
Outer core radius, R_c	340-410 km
Inner core radius, R_i	--
Surface gravitational acceleration, g	1.63 m s^{-2}
Thermal diffusivity, κ	10^{-6} m^2 s^{-1}
Latent heat, L	$6x10^5$ J mol^{-1}
Core thermal conductivity, k_c	50 W·m^{-1}·K^{-1}
Crust thermal conductivity, k_{cr}	2 - 4 W·m^{-1}·K^{-1}
Thermal expansion of mantle, α	$2.3x10^{-5}$ K^{-1}
Thermal expansion of core, α_c	$9x10^{-5}$ K^{-1}
Temperature difference, ΔT	1660 K
Initial CMB temperature, T_c	1610 ^{o}C
Upper mantle density, ρ_0	3300 kg m^{-3}
IBC density, ρ_{IBC}	3450-3700 kg m^{-3}
Lower mantle density, ρ_{LM}	3410 kg m^{-3}
Crust density, ρ_c	2900 kg m^{-3}
Core density, ρ_{core}	7800 kg m^{-3}
Mantle specific heat, C_p	1200 J·kg^{-1}·K^{-1}
Core specific heat, C_{pc}	800 J·kg^{-1}·K^{-1}
Reference viscosity, η_0	$5x10^{19}$-10^{21} Pa·s
Core sulfur content, S	5%

Supplementary Table S3. Additional Tests of Model Input Parameters Relative to Runs 1 - 11 in Table 1.

Model	Model Input			Internal Test	Melt Vol. (% of crust)	FWHM (Myrs)	Mag. Timing (Myrs)
	IBC (km)	η contrast	Ref. η (Pa · s)				
**Run 1a**	**30**	**10^{-1}**	**$5x10^{20}$**	Thermal conductivity of crust was decreased from 4 W m^{-1} K^{-1} (Run 1) to 2W m^{-1} K^{-1}	8	71	156
**Run 1H**	**30**	**10^{-1}**	**$5x10^{20}$**	Initial temperature hotter than Run 1 (supplementary Fig. S8)	57	16	58
**Run 3C**	**30**	**10^{-2}**	**$5x10^{20}$**	Initial temperature colder than Run 3 (supplementary Fig. S7)	0.04	NA	NA
**Run 3C_i**	**30**	**10^{-2}**	**$5x10^{19}$**	Initial temperature colder than Run 3 (supplementary Fig. S7)	0.03	2	5
**Run 6H**	**50**	**10^{-2}**	**10^{21}**	Initial temperature hotter than Run 6 (supplementary Fig. S8)	23	10	29
